# The Impact of the Covid-19 Pandemic on Disgust Sensitivity

**DOI:** 10.3389/fpsyg.2020.600761

**Published:** 2021-01-20

**Authors:** Richard J. Stevenson, Supreet Saluja, Trevor I. Case

**Affiliations:** Department of Psychology, Macquarie University, Sydney, NSW, Australia

**Keywords:** disgust, infection, avoidance, hand hygiene, germ aversion

## Abstract

There have been few tests of whether exposure to naturalistic or experimental disease-threat inductions alter disgust sensitivity, although it has been hypothesized that this should occur as part of disgust’s disease avoidance function. In the current study, we asked Macquarie university students to complete measures of disgust sensitivity, perceived vulnerability to disease (PVD), hand hygiene behavior and impulsivity, during Australia’s Covid-19 pandemic self-quarantine (lockdown) period, in March/April 2020. These data were then compared to earlier Macquarie university, and other local, and overseas student cohorts, to determine if disgust sensitivity and the other measures, were different in the lockdown sample. The most consistent finding in the lockdown sample was of higher core disgust sensitivity (Cohen’s *d* = 0.4), with some evidence of greater germ aversion on the PVD, and an increase in hand and food-related hygiene, but with little change in impulsivity. The consistency with which greater core disgust sensitivity was observed, suggests exposure to a highly naturalistic disease threat is a plausible cause. Greater disgust sensitivity may have several functional benefits (e.g., hand and food-related hygiene) and may arise implicitly from the threat posed by the Covid-19 pandemic.

## Introduction

The idea that disgust serves a disease avoidance function has been suggested by several authors and by a number of empirical findings (e.g., Curtis et al., 2004; Oaten et al., 2009). In an examination of potential hypotheses to test the disease avoidance account of this emotion, Oaten et al. (2009) describe in hypothesis 7 how vulnerability to disease should affect disgust, and in particular, how disease threat should result in greater disgust sensitivity. As we outline further below, there have been relatively few tests of this and related hypotheses. Moreover, there have been none using what is probably the most powerful test, namely a naturalistic disease induction (i.e., exposure to a real-world pandemic of infectious disease; Tunnel, 1977; Fernandez-Dols and Civelli, 2013). The aim of the current study is to examine if the Covid-19 pandemic alters participants disgust sensitivity - alongside related measures - by contrasting responses obtained during the pandemic period with responses from comparable previous participant cohorts.

A number of laboratory induction studies have manipulated disease threat by exposing participants to pictures of sick people (e.g., Mortensen et al., 2010; Murray et al., 2013), people sneezing and coughing (Lee et al., 2010), descriptions of migrants who have come from countries believed to harbor more or less infectious disease (Faulkner et al., 2004), and by getting participants to describe a time when they felt especially vulnerable to disease (e.g., Murray and Schaller, 2012). All of these studies obtained shifts in attitudes, intentions or behaviors, consistent with greater disease avoidance (e.g., reduced extroversion, greater ethnocentricity etc.). These findings indicate that a range of behaviors and dispositions that should aid disease avoidance are increased and so lend indirect support to the idea that other related systems too, such as disgust sensitivity, should also be increased. However, there have been surprisingly few tests of this idea.

One approach has been to see if prior illness might up-regulate disgust sensitivity. Mechanistically, there are at least two ways this might happen. In the first, the biological immune system may act to increase disease avoidant behavior, including disgust. Miller and Maner (2011) tested whether recent illness increased attention to disfigured faces and avoidance of disfigured people and found that it did. They suggest an increase in disease avoidant behavior following illness is not mediated by conscious disease-related knowledge as the effects were independent of current disease concerns (measured using the Perceived Vulnerability to Disease [PVD] questionnaire; Duncan et al., 2009). Two studies using disgust sensitivity have been motivated by this type of approach. Stevenson et al. (2009b) examined the relationship between frequency and recency of common infectious diseases, disgust, and contamination sensitivity. Their results suggested that frequent illness led to heightened contamination sensitivity, which combined with disgust sensitivity, led to fewer illnesses via enhanced behavioral avoidance. However, a further study by de Barra et al. (2014) examining the same hypothesis in a Bangladeshi sample, found no link between disgust sensitivity and illnesses. In addition, Miller and Maner’s (2011) findings have also not been replicated (Tybur et al., 2020).

Another approach with the same biological motivation, has been to compare groups of participants who differ in disease risk and see if they also differ in disgust sensitivity. Two studies have adopted this approach. Fessler et al. (2005) examined for heightened disgust sensitivity in the first trimester of pregnancy, when the foetus is most vulnerable to infection. They found heightened disgust sensitivity in the first trimester, consistent with the increased disease risk – but not all studies have replicated this finding either (Jones et al., 2018). Oaten et al. (2017) used a conceptually similar approach, by contrasting people with rheumatoid arthritis who experience more infections (and have higher death rates from them too) with a control sample who did not have this disease. While people with rheumatoid arthritis had highly elevated scores on both subscales of the PVD relative to controls, they did not differ at all in disgust sensitivity, having almost identical means to controls.

Presumably, the circumstances under which we might best expect disgust sensitivity to increase, would be when exposed to a highly salient disease threat. There have been a number of studies that have utilized such naturalistic disease threat inductions, however, they have instead focused on fear of contracting the disease in question, and the predictors of this fear. This approach has been employed for Zika virus (Blakey and Abramowitz, 2017), Ebola (Blakey et al., 2015), and Swine flu (Brand et al., 2013; Wheaton et al., 2012). Interestingly, in each of these studies, contamination sensitivity (in all) and disgust sensitivity (in 3/4) were significant correlates of fear of contracting these various infectious diseases. Relatedly, Fan and Olatunji (2013) also reported that more general health anxiety was significantly related to disgust sensitivity. So, while we do not know if a naturalistic disease induction might affect disgust sensitivity, disgust sensitivity does seem to be implicated in the fear of contracting such diseases and disease fear more generally.

In the current study, our primary focus was on disgust sensitivity. As we wanted to compare our current cohort to previous cohorts that were demographically similar, we used the original 32 item Disgust Scale (DS; Haidt et al., 1994), but only utilized the 27 questions and three sub-scale scores of the revised version of this survey (DS-R; Olatunji et al., 2008, 2009). A further reason for using the DS is that it remains the only self-report disgust sensitivity measure to have been behaviorally validated (Rozin et al., 1999). Two other conceptually related measures were also included. The first was the Hygiene Behavior Inventory, which is a validated and reliable measure to assess multiple aspects of hygiene behavior, including hand washing (Stevenson et al., 2009a,b). This was used because a recent study of people in Croatia undertaken during the Covid-19 self-quarantine (lockdown) period, found large and highly significant increase in safety behaviors, including hand-hygiene (Korajlija and Jokic-Begic, 2020). Logically, we would expect hand-hygiene to increase, and thus to exceed those reported in the past. The second measure was the PVD (Perceived Vulnerability to Disease questionnaire; Duncan et al., 2009). This measure has been widely used to assess perceptions of disease threat and has been used in several related studies (e.g., Oaten et al., 2017). Moreover, much of the reasoning that would suggest that increased disease salience might drive increased disgust sensitivity would also presumably apply to perceived vulnerability to disease. Exactly this prediction was born out in a study that emerged after ours was completed, which found in a United States sample that perceived Covid-19 threat was linked to with higher PVD scores (Makhanova and Shepherd, 2020). The authors also found differences on the two PVD subscales, with the Germ aversion subscale more linked to behavioral disease avoidance and Perceived infectability more linked to disease vigilance. Together, the clear prediction would be of increased PVD scores.

In addition to these main measures, we also collected some other information. First, we included the Barratt Impulsiveness scale (BIS; Spinella, 2007). This was completed as we had no grounds to think that impulsivity would change in response to the Covid-19 pandemic as it is a relatively stable and heritable trait (e.g., Anokhin et al., 2015) and one which is generally negatively correlated with health behaviors (e.g., Duckworth and Kern, 2011). As we had collected BIS data at around the same time as the DS, PVD, and HBI, and in very similar samples, the BIS would serve as a test of any general heightening of response tendencies on survey instruments. Second, we also obtained basic demographic information, namely age and gender, as both are known to moderate disgust sensitivity (e.g., Haidt et al., 1994; Druschel and Sherman, 1999; Al-Shawaf et al., 2018). Participants were also asked whether they were ill now, whether they had been recently ill, and their general health status, on the basis that these variables might also modify responding (e.g., Prokop et al., 2010). The survey was undertaken during Australia’s lockdown period, and the studies relationship to the timeline of events is presented in Table 1. The date on which participants completed the survey was also used as a variable, on the basis that the further into the lockdown period the survey was completed, the more intense and saturated (i.e., media coverage, large change in routines of work, study and socialization, etc.) were peoples experience of the pandemic.

**TABLE 1:** Covid-19 timeline in Australia, prior to and during the survey period.

**Date**	**Event**
25/1/20	First Australian Covid-19 infection identified; China travel alert issued
3/2/20	Australians evacuated from Wuhan to quarantine on Christmas island
5/2/20	Mandatory quarantine for arrivals from China
11/2/20	13 cases now identified
1/3/20	First Covid-19 death
3/3/20	Panic buying starts in supermarkets
7/3/20	73 cases identified, 2 deaths
11/3/20	WHO declares global pandemic
15/3/20	298 cases identified, 5 deaths; Ban on gatherings of 500+ people
16/3/20	Macquarie University stops face-to-face teaching; Foreign arrivals must self-isolate; Shopping limits imposed at supermarkets
17/3/20	All international travel banned
18/3/20	Ban on gatherings of 100+
20/3/20	875 cases identified, 7 deaths; Social distancing rules enacted
23/3/20	Lock-down starts; Macquarie University campus closed; Schools, all entertainment venues, gyms, sports venues, many shops closed
25/3/20	2432 cases identified, 9 deaths; State borders closed
29/3/20	All Australians instructed to stay home unless in vital employment
1/4/20	4864 cases identified, 21 deaths
**23/4/20**	**Survey starts**; 6661 cases identified, 75 deaths
30/4/20	6762 cases identified, 92 deaths
2/5/20	Large out-break in Victoria
15/5/20	First easing of restrictions in NSW, with up to 10 patrons in restaurants
**30/5/20**	**Survey ends**

## Materials and Methods

### Participants

Three-hundred and twenty-two Macquarie University undergraduate psychology students started the survey and 310 successfully completed it for course credit. The survey was open from 23rd April 2020 and closed on the 30th May 2020. The survey opening date was around 3 months after the Covid-19 pandemic started to dominate news media in Australia (see Table 1 for timeline) and data was collected during the most intense phase of the pandemic, with Australia in lockdown for the study period. We refer to this sample as MU20 (i.e., data collected at Macquarie University in 2020).

The study protocol was approved by Macquarie University human ethics committee. Participants were informed at the start of the survey that the aim was to study relationship between emotion and behavior, and that they would be completing various questionnaires relating to disgust, perceptions of threat, hygiene and impulsivity – wording of the aim was vague and based on prior information statements used in collecting these sorts of data at Macquarie University. Participants were informed that completion of the survey indicated consent to use their data. At the end of the survey, participants were presented with a written debrief about the study’s primary aim and they were asked not to disclose this to other students.

### Comparison Samples

Seven comparison samples were used to establish if the MU20 sample might report alterations in the various study measures. Four of the comparison samples were collected at Macquarie University, one during 2008, one during 2009, one during 2010 and one during 2014. All four were completed wholly or mainly (more below) on first year psychology students using on-line data collection with Qualtrics. We detail each sample in turn.

The 2008 Macquarie sample (MU08), formed Study 5 of Stevenson et al. (2009a, b). Study 5 was composed primarily of first-year undergraduates, alongside participants from the university and local community. These participants completed online, the Hygiene Behavior Inventory (HBI), the Disgust Scale (original 32 item version), the Perceived Vulnerability to Disease questionnaire, alongside other measures (Mini-marker, Padua contamination index). The aim of the study was to test the construct validity of the HBI.

The 2009 Macquarie sample (MU09), is from an unpublished survey undertaken by author TIC, exploring contamination beliefs using the vignette based Rozin ‘sweater task’ (i.e., would you wear a sweater who had been worn by…). First-year undergraduates completed the Rozin ‘sweater task’ and then the PVD and the Disgust Scale (original 32 item version), as part of their course requirements.

The 2010 Macquarie sample (MU10), was also an unpublished survey undertaken by TIC, to explore a vignette-based measure of contamination, using product choices by stigma targets to see if this would affect hypothetical purchase decisions. First-year undergraduates completed this task and then the PVD and the Disgust Scale (original 32 item version), as part of their course requirements.

The 2014 Macquarie sample (MU14), formed the sample for Study 1 of Lumley et al., 2016, exploring the relationship between diet and impulsivity. Participants completed online the short form Barratt Impulsiveness Scale (BIS), a brief food frequency measure and demographic variables.

The 2009 University of Western Australia (UWA09) comparison, which consisted of just means and SDs for the Disgust Scale (original 32 item version), gender distribution and age, was obtained from Tables 1, 2 from Olatunji et al. (2009). This study reports samples from 8 different countries as part of a validation of the revised DS. We selected the Australian sub-study to provide a further Australian student sample from another university.

**TABLE 2:** Correlation (Pearson’s) between the main measure totals and scale scores (5% critical alpha = ± 0.11) for the current dataset.

**Scales**	**2.**	**3.**	**4.**	**5.**	**6.**	**7.**	**8.**	**9.**	**10.**	**11.**	**12.**	**13.**	**14.**	**15.**	**16.**	**17.**
**Subscales**																
1. DS-R Total_1_	0.86	0.80	0.67	0.49	0.58	0.18	0.50	0.47	0.22	0.34	0.35	0.17	−0.06	−0.01	−0.11	−0.01
2. Core		0.47	0.44	0.44	0.52	0.16	0.47	0.42	0.24	0.29	0.34	0.18	0.05	−0.01	−0.13	−0.01
3. Animal			0.35	0.31	0.34	0.15	0.27	0.26	0.09	0.26	0.19	0.05	0.04	0.04	−0.01	0.06
4. Contamination				0.42	0.54	0.10	0.48	0.50	0.20	0.24	0.32	0.18	−0.17	−0.13	−0.15	−0.12
5. PVD Total_2_					0.82	0.76	0.51	0.49	0.24	0.24	0.44	0.14	−0.09	−0.09	−0.12	−0.02
6. Germ aversion						0.28	0.62	0.61	0.25	0.30	0.48	0.21	−0.21	−0.18	−0.21	−0.10
7. Perceived infectability							0.17	0.15	0.12	0.07	0.20	−0.00	0.07	0.05	0.03	0.08
8. HBI Total_3_								0.88	0.51	0.56	0.74	0.45	−0.24	−0.17	−0.24	−0.15
9. General hygiene									0.27	0.42	0.57	0.21	−0.19	−0.16	−0.17	−0.12
10. Household hygiene										0.21	0.32	0.15	−0.20	−0.12	−0.23	−0.12
11. Food-related hygiene											0.28	0.08	−0.09	−0.02	−0.10	−0.08
12. Hand hygiene technique												0.15	−0.18	−0.10	−0.25	−0.06
13. Personal hygiene													−0.12	−0.09	−0.08	−0.12
14. BIS Total_4_														0.81	0.71	0.82
15. Motor															0.32	0.56
16. Non-planning																0.37
17. Attention																

*_1_ Disgust Scale-Revised. _2_ Perceived Vulnerability to Disease. _3_ Hygiene Behavior Inventory. _4_ Barratt Impulsiveness Scale.*

The 2008 Fordham University (USA08) comparison, again consisting of means and SDs for the Disgust Scale (original 32 item version), gender distribution and age, was obtained from Olatunji et al. (2008). Olatunji et al. (2008) report four studies in total aimed at developing and validating a revision of the DS, and we selected just the Fordham sample (Study 3) as it was the largest and most comparable in gender distribution.

The 2009 University of British Columbia (UBC09) comparison, consisted of means and SDs for the PVD, gender distribution and age, reported as part of the development and validation of this scale (Duncan et al., 2009). A smaller Dutch sample was also included, but we selected the larger UBC sample both because of its size and due to the greater cultural and linguistic similarity between Canada and Australia.

### Measures

The Disgust Scale (DS) was administered in its original 32-item version (Haidt et al., 1994), to provide the same question context as the various comparison samples described above. We then used the subset of 25 items identified in Olatunji et al.’s (2008) revision (DS-R), and its three resultant subscales (Core, Animal reminder, Contamination), alongside the total score. The DS-R has good overall reliability (alpha > 0.8), with adequate reliability for the subscales.

The Perceived Vulnerability to Disease (PVD) questionnaire is a 15-item measure assessing participants perceived susceptibility to catching disease and their aversion to pathogens. The scale has good reliability, as do each of its two subscales (Germ aversion and Perceived infectability; alpha > 0.74).

The Hygiene Behavior Inventory (HBI) is a 23-item measure that asks participants about several domains of hygiene-related behavior (Stevenson et al., 2009a,b). The scale has 5 sub-scales, measuring General hygiene (8 items, 6 on hand washing situations), Household hygiene (3 items on household cleaning), Food-related hygiene (3 items on preparing food), Hand hygiene technique (5 items on knowledge about appropriate means of washing hands) and Personal hygiene (4 items on clothing change and bathing habits). Overall reliability for the scale is good (alpha = 0.85), and subscale alphas range from good to adequate (0.82–0.67). Responses on the HBI predict hand hygiene behavior and reported infection rates for common illnesses (Stevenson et al., 2009a,b).

The short form Barratt Impulsiveness scale (BIS) is a 15-item measure that assesses impulsivity (Spinella, 2007). The scale has three sub-scales (Motor, Non-planning and Attention), with good overall reliability (alpha > 0.79; sub-scale alphas not reported).

### Procedure

Participants completed the online Qualtrics survey in a fixed order, undertaking the DS-R, then the PVD, HBI and the BIS. The date the survey was completed was also recorded. After completing the questionnaires, participants were asked to report their age and gender, their current health [five point category scale from 1 (Poor) to 5 (Excellent)], and whether they had been ill in the last month, week or were currently ill (in each case Yes, No, Unsure). Six check questions were randomly interspersed throughout the survey to ensure that participants were paying attention and not answering in a repetitive manner. All of these were correctly answered by the participants. A brief one paragraph debriefing was presented on completion of the survey.

### Analysis

Apart from illness recency and general health, the remaining data were normally distributed and suitable for parametric testing. Bivariate relationships were established using Pearson’s for normal data, with Spearman’s for correlations involving non-normal variables. To check differences between samples in age and gender distribution, independent *t*-tests were used for the former and chi-squared for the latter. Two main analysis approaches were then used. The first, where we had access to raw data, used Multivariate Analysis of Covariance (MANCOVA), with Sample (MU20 vs. comparison) and Gender as between subject factors, sub-scale scores as the dependent variables and age as the covariate. Both the outcome of the multivariate tests (i.e., Sample, Gender, Gender by Sample) and the univariate effects for each sub-scale are reported. The second approach was employed for comparisons where we only had access to means and standard deviations. Here we used independent t-tests on each sub-scale score.

## Results

### Analysis of Just the MU20 Sample

Correlations between the measures for the MU20 sample obtained during the Covid-19 lockdown are detailed in Table 2. Consistent with the previous literature, greater disgust sensitivity (DS-R) was positively associated with greater perceived vulnerability to disease (PVD), and especially so for its germ aversion subscale. Greater disgust sensitivity and greater perceived vulnerability to disease were both correlated with greater self-reported propensity for hygiene behaviors (HBI). Better self-reported hygiene, greater disgust sensitivity and greater perceived vulnerability to disease were all weakly linked to lower levels of self-reported impulsivity (BIS).

The MU20 sample were asked about their current general health status, with the modal response being good (48.1%). MU20 participants were also asked about recent illness, with 21.6% reporting having been ill in the last month, 9.4% in the last week and 5.5% while completing the survey. We also recorded when during the data collection period the survey was completed – testing order. All of these variables were then correlated (Spearman’s rho) with the total scores of the main measures, partialling out age and gender. The correlations are presented in Table 3. The recent illness variables were unrelated to any of the main measures. However, better general health was linked to both lower reported perceived vulnerability to disease (PVD) and to less impulsive behavior (BIS).

**TABLE 3:** Correlation (Spearman) between testing order, general health, and recent illness, and the total scores for the main measures, partialling out age and gender, for the current dataset.

**Variable**	**Testing order**	**General health**	**Unwell past month**	**Unwell past week**	**Unwell now**
DS-R_1_	0.14*	−0.05	0.02	0.10	0.02
PVD_2_	0.12*	−0.18*	−0.06	−0.06	−0.03
HBI_3_	0.03	0.07	−0.02	−0.01	0.05
BIS_4_	0.06	−0.23*	0.03	0.00	−0.02

*p < 0.05. _1_ Disgust Scale-Revised. _2_ Perceived Vulnerability to Disease. _3_ Hygiene Behavior Inventory. _4_ Barratt Impulsiveness Scale.

The later participants completed the survey (i.e., the further into the lockdown period of the pandemic) the higher their scores on both disgust sensitivity (DS-R) and on the PVD. For the DS-R, the mean score for participants who completed data collection in the first half of the survey collection period was 14.5 (SD = 4.2), increasing to a mean of 15.6 (SD = 4.1) in the second half of the survey collection period – a 4.4% increase. For the PVD, the comparable change in means was from 3.7 (SD = 0.9) to 3.9 (SD = 0.8), representing a 2.9% increase.

### Comparison of the MU20 Sample to Other Student Samples for Disgust Sensitivity

Descriptive data for the MU20 sample and the comparison samples for disgust sensitivity are presented in Table 4. Five comparison samples were available, three from Macquarie (MU10, 09, 08), one from the University of Western Australia (UWA09) and one from Fordham University (USA08) in the United States. The MU20 sample was significantly younger - by around 1 year - than the other Macquarie samples (*p* < 0.016), hence our use of age as a covariate in the analyses using raw data (i.e., MU10, 09, 08). The UWA09 sample was also significantly younger than the MU20 sample by a similar amount (*p* = 0.0009) but the USA08 sample did not differ in age. As we did not have raw data for these last two comparisons, age difference could not be corrected for the UWA09 sample. For the proportion of males to females, only the MU10 sample differed from the MU20 (*p* = 0.044; all other *p*’s > 0.21), but as gender could potentially moderate some of the psychological effects of Covid-19 pandemic, it was included as an independent variable in the analyses using raw data (i.e., MU10, 09, 08).

**TABLE 4:** Sample details for the analyses of the Disgust Scale-Revised (DS-R).

**Study (Name)**	***n* = **	**Mean**	**DS-R subscales means (SDs)**		
**Year**	**(%female)**	**Age (SD)**	**Core**	**Animal**	**Contamination**
**Macquarie university students during CV19 lockdown (MU20)**					

2020_1_	310 (75.2)	19.8 (3.8)	8.19 (2.14)	5.01 (1.94)	1.85 (1.19)

**Macquarie university students in previous years (MU10, 09 and 08)**					

2010_2_	467 (68.7)	20.8 (5.3)	7.40 (2.39)	4.86 (1.66)	1.83 (1.19)
2009_3_	632 (73.7)	20.6 (5.2)	7.30 (2.43)	4.98 (1.68)	1.69 (1.16)
2008_4_	507 (74.6)	21.0 (5.2)	7.40 (2.37)	4.11 (1.80)	1.78 (1.19)

**Australian (non-Macquarie) university students in previous years (UWA09)**					

2009_5_	646 (71.5)	18.9 (4.5)	6.60 (2.28)	3.92 (2.00)	0.95 (0.95)

**American university students in previous years (USA08)**					
2008_6_	363 (74.0)	20.0 (1.6)	7.32 (2.64)	5.36 (2.08)	3.55 (1.45)

*_1_ Macquarie university psychology undergraduate sample collected during the Covid-19 lockdown and university closure period. _2_ Macquarie university psychology undergraduate sample collected in 2010 by author TIC. _3_ Macquarie university psychology undergraduate sample collected in 2009 by author TIC. _4_ Macquarie university psychology undergraduate sample collected as part of the American Journal of Infection Control study published by RS and TC. _5_ Australian arm (UWA: University of Western Australia) of Olatunji et al., 2009. _6_ Study 3 Olatunji et al., 2008, collected from undergraduates at Fordham university, New York.*

**FIGURE 1:**
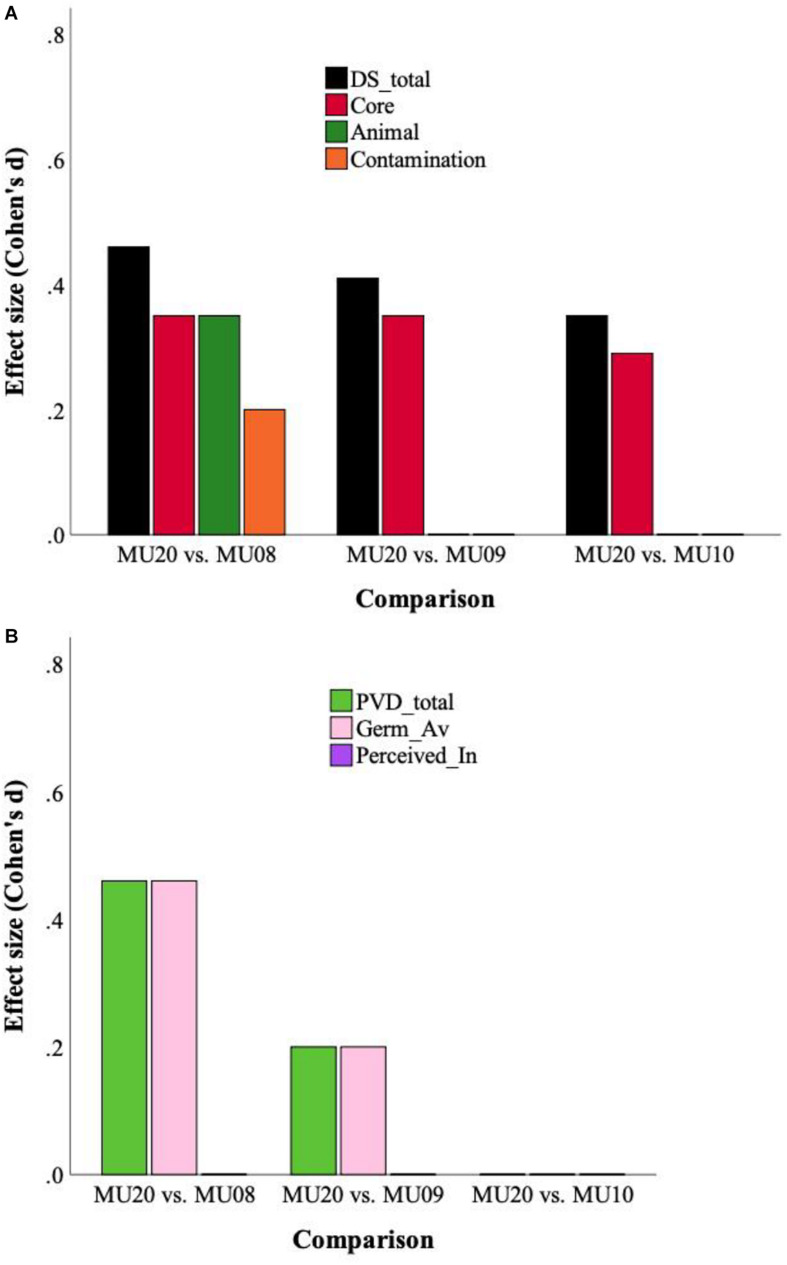
**(A)** Effect sizes for Disgust Sensitivity (DS) total score and subscales, for the three raw data analyses. **(B)** Effect sizes for Perceived Vulnerability to Disease (PVD) and subscales (Germ aversion, Perceived infectability), for the three raw data analyses.

The two different sets of analyses are presented in Table 5. The raw data analyses, using MANCOVA (Sample, Gender; Age as covariate) with the three subscales as dependent variables, revealed significant effects of Sample and of Gender, for each of the three analyses, with an interaction between Sample and Gender for just the MU08 comparison [here the gender difference for Contamination was larger in the MU08 sample (*M* = 0.9), than in the MU20 sample (*M* = 0.4)]. In all three analyses, disgust sensitivity was higher in the MU20 sample, and as would be expected, higher in women across all samples. Univariate effects for Sample are also reported in Table 5. Only Core disgust was significantly higher in all three comparisons. The raw data analyses are illustrated using effect size in Figure 1A.

**TABLE 5:** Comparison of the current sample with previous student samples for the Disgust Scale-Revised.

**Comparison**						

**Statistical methods and outcomes**						

**MANCOVA (age as covariate)**	**Sample (S)**	**Gender (G)**	**Gender × Sample (G × E)**	**Univariate effects for Sample for Core (C)**	**Univariate effects for Sample for Animal (A)**	**Univariate effects for Sample for Contamination (N)**
**MU20 vs. MU10**						
	S: *F*_3,770_ = 6.60*, η^2^ = 0.03	G: *F*_3,770_ = 53.95*, η^2^ = 0.17	G**×** S: *F*_3,770_ = 0.17, η^2^ = 0.00	C: *F*_1,772_ = 12.97*, η^2^ = 0.02	A: *F*_1,772_ = 0.04, η^2^ = 0.00	N: *F*_1,772_ = 0.07, η^2^ = 0.00
**MU20 vs. MU09**						
	S: *F*_3,935_ = 12.15*, η^2^ = 0.04	G: *F*_3,935_ = 57.87*, η^2^ = 0.16	G × S: *F*_3,935_ = 0.39, η^2^ = 0.00	C: *F*_1,937_ = 27.20*, η^2^ = 0.03	A: *F*_1,937_ = 0.12, η^2^ = 0.00	N: *F*_1,937_ = 2.32, η^2^ = 0.00
**MU20 vs. MU08**						
	S: *F*_3,810_ = 12.71*, η^2^ = 0.05	G: *F*_3,810_ = 56.34*, η^2^ = 0.17	G × S**: *F*_3,810_ = 4.30*, η^2^ = 0.02	C: *F*_1,812_ = 25.01*, η^2^ = 0.03	A: *F*_1,812_ = 27.68*, η^2^ = 0.03	N: *F*_1,812_ = 4.96*, η^2^ = 0.01

**Independent *t*-tests (only Ms and SDs available)**						
	
	**Core (C)**	**Animal (A)**	**Contamination (N)**			

**MU20 vs. UWA09**						
	C: t_954_ = 10.29*, *r*^2^ = 0.10	A: t_954_ = 7.96*, *r*^2^ = 0.06	N: t_954_ = 12.59*, *r*^2^ = 0.14			
**MU20 vs. USA08**						
	C: t_671_ = 4.64*, *r*^2^ = 0.03	A: t_671_ = −2.24*, *r*^2^ = 0.01	N: t_671_ = −16.45*, *r*^2^ = 0.29			

*p < 0.05. **Univariate effects revealed that the Gender x Sample interaction was only significant for N, *F*_1_,_812_ = 9.02*, η^2^ = 0.01.

The two final samples, UWA09 and USA08 were analyzed by independent t-tests, one for each subscale (see Table 5). For the UWA09 comparison, Core, Animal reminder and Contamination disgust sensitivity were higher in the MU20 sample. For the USA08 comparison, Core disgust was greater in the MU20 sample, but the Fordham students reported significantly higher Animal reminder and Contamination disgust sensitivity. In sum, the consistent finding from this set of analyses was of higher reported core disgust sensitivity in the MU20 sample.

### Comparison of the MU20 Sample to Other Student Samples for Perceived Vulnerability to Disease

Relevant descriptive data for the MU20 sample and the comparison samples for the PVD questionnaire are presented in Table 6. Four comparison samples were available, three from Macquarie (MU10, 09, 08), and one from Canada (UBC09). As noted, the MU20 sample was significantly younger - by around 1 year - than the three Macquarie samples (an age comparison could not be made for the UBC sample, but it too is around 1 year older than the MU20 sample). The MU10 sample also had slightly but significantly fewer women and more men than the MU20 sample. There were no differences in gender distribution for the other comparison samples.

**TABLE 6:** Sample details for the analyses of the Perceived Vulnerability to Disease (PVD) Questionnaire.

**Questionnaire**					
**Study (Name)**	**n =**	**Mean**	**PVD – Means (SDs)**		
**Year**	**(%female)**	**Age (SD)**	**Total**	**Germ**	**Perceived**
			**score**	**Aversion**	**infectability**
**Macquarie university students during CV19 lockdown (MU20)**					
2020_1_	310 (75.2)	19.8 (3.8)	3.83 (0.83)	4.03 (1.02)	3.59 (1.06)
**Macquarie university students in previous years (MU10, 09, and 08)**					
2010_2_	467 (68.7)	20.8 (5.3)	3.75 (0.93)	3.88 (1.09)	3.61 (1.30)
2009_3_	632 (73.7)	20.6 (5.2)	3.73 (0.89)	3.81 (1.02)	3.63 (1.27)
2008_4_	507 (74.6)	21.0 (5.2)	3.49 (0.90)	3.44 (1.03)	3.56 (1.30)
**Canadian university students in previous years (UBC09)**					
2009_5_	1332 (75.6)	20.8_5_	3.67 (1.07)	3.81 (1.02)	3.52 (1.12)

*_1_ Macquarie university psychology undergraduate sample collected during the Covid-19 lockdown and university closure period. _2_ Macquarie university psychology undergraduate sample collected in 2010 by author TIC. _3_ Macquarie university psychology undergraduate sample collected in 2009 by author TIC. _4_ Macquarie university psychology undergraduate sample collected as part of the American Journal of Infection Control study published by RS and TC. _5_ Canadian university students at the University of British Columbia reported in Duncan et al., 2009. _5_ No SD provided.*

Two sets of analyses were completed, which are presented in Table 7. The first used MANCOVA, and revealed significant effects of Gender in all cases, no Sample by Gender interactions, and effects of Sample for two out of the three comparisons. PVD scores were higher overall for the MU20 sample in the MU09 and MU08 comparisons. Females consistently scored higher than males. Univariate effects for sample are also reported in Table 7. There were no univariate effects for the MU10 comparison, but for MU09 and MU08, the Germ Aversion subscale, but not the Perceived Infectability subscale, was higher in the MU20 sample. The raw data analyses are illustrated using effect size in Figure 1B.

**TABLE 7:** Comparison of the current sample with previous student samples for the Perceived Vulnerability to Disease Questionnaire.

**Comparison**					
	**Statistical methods and outcomes**				

**MANCOVA (age as covariate)**	**Sample (S)**	**Gender (G)**	**Gender × Sample (G × E)**	**Univariate effects for Sample for Germ aversion (GA)**	**Univariate effects for Sample for Infectability (PI)**
**MU20 vs. MU10**					
	S: *F*_2,771_ = 1.10, η^2^ = 0.00	G: *F*_2,771_ = 18.19*, η^2^ = 0.05	G**×** S: *F*_2,771_ = 0.08, η^2^ = 0.00	GA: *F*_1,772_ = 1.28, η^2^ = 0.00	PI: *F*_1,772_ = 0.49, η^2^ = 0.00
**MU20 vs. MU09**					
	S: *F*_2,936_ = 3.54*, η^2^ = 0.01	G: *F*_2,936_ = 25.84*, η^2^ = 0.05	G × S: *F*_2,936_ = 1.13, η^2^ = 0.00	GA: *F*_1,937_ = 6.64*, η^2^ = 0.01	PI: *F*_1,937_ = 0.01, η^2^ = 0.00
**MU20 vs. MU08**					
	S: *F*_2,811_ = 21.84*, η^2^ = 0.05	G: *F*_2,811_ = 17.69*, η^2^ = 0.04	G × S: *F*_2,811_ = 0.97, η^2^ = 0.00	GA: *F*_1,812_ = 42.61*, η^2^ = 0.05	PI: *F*_1,812_ = 0.11, η^2^ = 0.00

**Independent t-tests (only Ms and SDs available)**					

	**Total (T)**	**Germ (GA),**		**Infectability (PI)**	

**MU20 vs. UBC09**					
	T: t_1640_ = 2.47*, *r*^2^ = 0.00	GA: t_1640_ = 3.42*, *r*^2^ = 0.01		PI: t_1640_ = 1.00, *r*^2^ = 0.00	

*^∗^*p* < 0.05.*

The final comparison used independent t-tests, one for each subscale (see Table 7). Relative to UBC09, the MU20 sample had a higher overall PVD score, and a higher Germ Aversion subscale score. There was no difference for the perceived infectability subscale. These analyses provide some evidence of an increase in germ aversion in the MU20 sample, noting that this subscale is far more strongly correlated with core disgust sensitivity than perceived infectability (see Table 2; Williams test comparison, *p* < 0.001).

### Comparison of the MU20 Sample to Another Student Sample for Hygiene Behavior

Relevant descriptive data for the MU20 sample and the comparison sample for the HBI questionnaire is presented in Table 8. The comparison sample was significantly older (by around 1 year), but with no difference in gender distribution.

**TABLE 8:** Sample details for the analyses of the Hygiene Behavior Inventory.

**Study (Name) Year**	***n* = **	**Mean**	**Hygiene Behavior Inventory – Means (SDs)**					
	**(%female)**	**Age (SD)**	**Total**	**General**	**Household**	**Food-related**	**Hand-hygiene**	**Personal**
			**score**	**hygiene**	**hygiene**	**hygiene**	**technique**	**hygiene**
**Macquarie university students during CV19 lockdown (MU20)**								
2020_1_	310 (75.2)	19.8 (3.8)	3.13 (0.37)	3.02 (0.57)	3.50 (0.56)	3.62 (0.54)	2.94 (0.45)	2.97 (0.53)
**Macquarie university students in previous years (MU08)**								
2008_2_	507 (74.6)	21.0 (5.2)	2.92 (0.38)	2.78 (0.52)	3.48 (0.61)	3.26 (0.71)	2.56 (0.53)	3.02 (0.54)

*_1_ Macquarie university psychology undergraduate sample collected during the Covid-19 lockdown and university closure period.)_2_ Macquarie university psychology undergraduate sample collected as part of the American Journal of Infection Control study published by RS and TC.*

The analyses are presented in Table 9. MANCOVA revealed significant effects of Sample and Gender, and a Sample by Gender interaction [here the gender difference for General hygiene was larger in the MU20 sample (*M* = 0.4), than for the MU08 sample (*M* = 0.1)]. Overall, self-reported hygiene scores were higher in the MU20 sample, and in women in both samples. Univariate effects for Sample are also reported in Table 9. Significant effects on three subscales were evident, General hygiene, Food-related hygiene and Hand-hygiene technique.

**TABLE 9:** Comparison of the current sample with previous student sample for the Hygiene Behavior Inventory.

**Comparison**								

	**Statistical method and outcome**							

**MANCOVA (age as covariate)**	**Sample (S)**	**Gender (G)**	**Gender × Sample (G × E)**	**Univariate effects for Sample for General hygiene (GH)**	**Univariate effects for Sample for Household hygiene (HH)**	**Univariate effects for Sample for Food-related hygiene (FRH)**	**Univariate effects for Sample for Hand-hygiene technique (HHT)**	**Univariate effects for Sample for Personal hygiene (PH)**
**MU20 vs. MU08**								
	S: *F*_5,808_ = 21.47*, η^2^ = 0.12	G: *F*_5,808_ = 12.84*, η^2^ = 0.07	G**×** S**: *F*_5,808_ = 2.67*, η^2^ = 0.02	GH: *F*_1,812_ = 15.99*, ^2^ = 0.02	HH: *F*_1,812_ = 0.00, η^2^ = 0.00	FRH: *F*_1,812_ = 36.97*, η^2^ = 0.04	HHT: *F*_1,812_ = 77.27*, η^2^ = 0.09	PH: *F*_1,812_ = 0.09, η^2^ = 0.00

*p < 0.05. **Univariate effects revealed that the Gender x Sample interaction was only significant for GH, *F*_1_,_812_ = 6.83*, η^2^ = 0.01.

### Comparison of the MU20 Sample to Another Student Sample for Impulsivity

Relevant descriptive data for the MU20 sample and the comparison sample for the BIS questionnaire is presented in Table 10. The MU14 comparison sample was the same age (*p* = 1) but had significantly more men than the MU20 sample (*p* < 0.01). The analyses are presented in Table 11. MANCOVA (Sample, Gender; Age as covariate), with the three subscales of the BIS as dependent variables, revealed only a significant effect of Gender, with women reporting slightly lower levels of impulsivity than men. Univariate effects for Sample are also reported in Table 11. A small univariate effect was present for the Attention subscale – this being reportedly poorer in the MU20 sample, but no effects on the Motor or Non-planning subscales were evident.

**TABLE 10:** Sample details for the analyses of the short form Barratt Impulsiveness Scale (BIS).

**Study (Name)**						
**Year**	***n*** = **(%female)**	**Mean Age (SD)**	**BIS – Means (SDs)**			
			**Total score**	**Motor**	**Non-planning**	**Attention**
**Macquarie university students during CV19 lockdown (MU20)**						

2020_1_	310 (75.2)	19.8 (3.8)	33.20 (6.76)	10.95 (2.94)	10.95 (2.81)	11.31 (2.88)

**Macquarie university students in previous years (MU14)**						

2014_2_	571 (66.7)	19.8 (4.6)	32.57 (6.76)	11.00 (2.97)	10.75 (2.96)	10.82 (2.97)

*_1_ Macquarie university psychology undergraduate sample collected during the Covid-19 lockdown and university closure period. _2_ Macquarie university psychology undergraduate sample (MU14) collected as part of the Personality and Individual Differences study published by RS.*

**TABLE 11:** Comparison of the current sample with a previous student sample for the short form Barratt Impulsiveness Scale (BIS).

**Comparison**						

	**Statistical method and outcomes**					
	
**MANCOVA (age as covariate),**	**Sample (S),**	**Gender (G)**	**Gender × Sample (G × E)**	**Univariate effects for Sample for Motor (M)**	**Univariate effects for Sample for Non-planning (NP)**	**Univariate effects for Sample for Attention (A)**
**MU20 vs. MU14**						

	S: *F*_3,874_ = 2.59, η^2^ = 0.01	G: *F*_3,874_ = 3.76*, η^2^ = 0.01	G**×** S: *F*_3,874_ = 0.04, η^2^ = 0.00	M: *F*_1,876_ = 0.02, η^2^ = 0.00	NP: *F*_1,876_ = 1.54, η^2^ = 0.00	A: *F*_1,876_ = 4.20*, η^2^ = 0.01

*p < 0.05.

## Discussion

The MU20 sample who completed the disgust sensitivity measure and other scales during Australia’s lockdown period of the Covid-19 pandemic, reported overall higher levels of disgust sensitivity than the Macquarie university comparison samples. When looking at the three subscales that constitute the DS-R, core disgust was consistently elevated (M Cohen’s *d* = 0.4), both in the three Macquarie university comparison samples and also relative to those of students from another Australian university and from an American university. There was no evidence that the presumed Covid-19 effect on core disgust was moderated by gender, although gender moderation was observed in one comparison, and then only for the contamination subscale. There was also some evidence that one of the two PVD subscales was elevated in the MU20 sample. While no changes were observed for the perceived infectability subscale, germ aversion was higher in the MU20 sample in 3 of the 4 comparisons (M Cohen’s *d* = 0.2). As would be expected, participants in the MU20 sample reported higher scores for hygiene behavior overall (Cohen’s *d* = 0.7), and on the subscales for hand-hygiene technique, general hygiene (6/8 items on hand washing occasions) and food-related hygiene. This is consistent with another Covid-19 study, which also found significant increases in safety behaviors, which included hand washing (Korajlija and Jokic-Begic, 2020). We also examined for changes in impulsivity, finding no overall effect, except for a single small univariate effect on the attention subscale (Cohen’s *d* = 0.2; poorer attention in the MU20 sample).

A significant concern in comparing data from one cohort to another, is whether any differences between the two arose from reasons other than the variable of interest (i.e., Covid-19). While no definitive answer to this concern can be given, there are several reasons to regard the observed differences as arising primarily from the Covid-19 pandemic. First, both authors RS and TC have worked at Macquarie University for the whole of the time period covered by the analyses. We are not aware of any major changes in demographics or sources of students into the university over the time periods covered here. Second, it is important to also identify the variables that were not reliably different when the samples were compared, especially given the power here to detect even small differences. The perceived infectability subscale of the PVD remained consistently similar, perhaps reflecting this measures sensitivity to personal infection history (i.e., the items pertain to belief that one will fall ill) rather than to perceptions of infection risk (e.g., Oaten et al., 2017). On the hygiene inventory (HBI), personal and household hygiene scores also remained stable. These subscales have no hand hygiene components, refer to frequency of clothing change and room cleaning, which might be expected to be least affected by a disease-related upswing in hygiene behavior. Reported impulsivity was also very similar, differing unexpectedly in only one subscale, with a small effect size. Third, the pattern of gender differences for all of the variables remained largely consistent across the cohorts, suggesting stability in this regard. Finally, a related issue concerns sample selection bias. We utilized all of the large undergraduate data sets we possess that included the DSQ, PVD, HBI or BIS, alongside data we could find from published reports that included subscale means, standard deviations and gender distributions, drawn from demographically, culturally and linguistically similar cohorts (i.e., undergraduates from English speaking countries).

Our contention is that the consistent effects observed for core disgust, and to some extent germ aversion, reflect reactions to the Covid-19 pandemic rather than some other unrelated cohort difference. On an individual level the pandemic involves exposure to unremitting media coverage of the pandemic (both reassuring, fear provoking and factual in content), physical and social isolation, potential loss of income, large alterations inbehavior (e.g., distancing, hand hygiene, home learning) and heightened vigilance to disease-relevant cues. We suggest that the import of these changes results in up-regulated disgust sensitivity and to some extent germ aversion. Both of these constructs are strongly related (Cohen’s *d* = 1.2) and both were also found to be increasing across the course of the survey period. This latter effect can be regarded as akin to a dose-response effect, with those completing the survey toward the end of the study period exposed to cumulatively more of the Covid-19 pandemic than those completing it early on.

One consideration is the functional import of increases in disgust sensitivity and relatedly, germ aversion. First, it is apparent from Table 2 that both disgust sensitivity and germ aversion are higher in individuals who report greater levels of hygiene behavior. Experimental tests suggest that disgust-based interventions both in the laboratory and real-world settings can produce increases in hand-hygiene behavior (Porzig-Drummond et al., 2009; Pellegrino et al., 2016). In addition, women in both laboratory and naturalistic settings wash their hands more frequently than men (e.g., Porzig-Drummond et al., 2009; Filion et al., 2011; Prokop et al., 2014). As we noted in this report and as widely documented elsewhere, women also report being more disgust sensitive than men (Al-Shawaf et al., 2018) and in addition, in the current study, women also reported higher rates of illness in the preceding week than men (15 vs. 4%). The combined consequence of these effects may be to increase the tendency to engage in hand-hygiene. A second consequence may relate to food. While reported increases in general hygiene and hand hygiene technique in the MU20 sample were predictable, as these measures primarily relate to hand washing, we also observed a robust increase in food-related hygiene too (Cohen’s *d* = 0.4) - yet this subscale has only one hand washing item. Disgust has often been conceptualized has having its phylogenetic roots in food avoidance (Rozin et al., 2016) and so a further consequence of increasing disgust sensitivity may be greater wariness around eating and food preparation. Third, as we noted at the start of the manuscript, disease inductions affect a wide variety of behaviors that are purported to improve disease avoidance (Faulkner et al., 2004; Lee et al., 2010; Prokop and Fancovicova, 2010; Murray and Schaller, 2012; Murray et al., 2013). While there has been some interest in identifying if the PVD serves to moderate these effects – and there is some evidence that it does (Murray et al., 2013) – it is not currently known if this also holds for disgust. If it does, then enhanced disgust sensitivity might also facilitate, these broader types of avoidant behavior.

A further question is *how* the various experiences that comprise the Covid-19 pandemic drive up disgust sensitivity and in particular *what* specific aspects of the experience might be responsible? As the average increase in disgust sensitivity would be quite small, it would presumably not be self-evident (in contrast to knowing that a pandemic was underway etc.), suggesting it might be an implicit change (or consistent with Pinker (1997), p383] - disgust as an intuitive microbiology). Finally, we also tentatively suggest that just as pain is more intense when it is perceived as threatening (e.g., a pain *may* mean irreparable tissue damage or just a bruise; Jackson et al., 2014), the same may also hold for disgust (Stevenson et al., 2019). It would seem reasonable to presume that the level of threat that people perceive during the Covid-19 pandemic would be far higher than normal and so this could in turn increase the intensity of disgust sensitivity. The arguably parallel finding in the pain literature is highly robust (Jackson et al., 2014).

In conclusion, we find that relative to earlier undergraduate cohorts - and assuming their similarity in most other regards – the MU20 sample who completed disgust sensitivity and other measures during the lockdown period of the Covid-19 pandemic, report higher disgust sensitivity, possibly greater germ aversion, an increase in safety behavior (hand washing), but with little change in impulsivity. We suggest that the putative increases in disgust sensitivity may have several functional benefits, and that the increase in disgust sensitivity arises implicitly from the threat of the Covid-19 pandemic.

## Data Availability Statement

The raw data supporting the conclusion of this article will be made available by the authors on request.

## Ethics Statement

The studies involving human participants were reviewed and approved by Macquarie University Human Research Ethics Committee. The patients/participants provided their written informed consent to participate in this study.

## Author Contributions

RS and TC conceived the study. RS analyzed the data and drafted the manuscript. SS undertook the data collection. TC and SS reviewed and revised the manuscript. All authors contributed to the article and approved the submitted version.

## Conflict of Interest

The authors declare that the research was conducted in the absence of any commercial or financial relationships that could be construed as a potential conflict of interest.
